# Health and Wellness Status Perception of Half-Marathon Runners: Influence of Age, Sex, Injury, and Training with Qualified Staff

**DOI:** 10.3390/ijerph17165649

**Published:** 2020-08-05

**Authors:** Estibaliz Romaratezabala, Daniel Castillo, Javier Raya-González, Josune Rodríguez-Negro, Irati Aritzeta, Javier Yanci

**Affiliations:** 1 Physical Education and Sport Department, Faculty of Education and Sport, University of the Basque Country, UPV/EHU, Basque Country 01007 Vitoria-Gasteiz, Spain; estibaliz.romaratezabala@ehu.eus (E.R.); josune.rodriguez@ehu.eus (J.R.-N.); irati.aritzeta@gmail.com (I.A.); javier.yanci@ehu.eus (J.Y.); 2 Society, Sports and Physical Exercise Research Group (GIKAFIT), University of the Basque Country (UPV/EHU), Basque Country 01007 Vitoria-Gasteiz, Spain; 3 Faculty of Health Sciences, Universidad Isabel I, 09003 Burgos, Spain; rayagonzalezjavier@gmail.com

**Keywords:** recreational athlete, running, endurance, musculoskeletal injury, women in sport

## Abstract

The aim of this study was to analyze the health and wellness status perception in amateur half-marathon runners according to sex, age, being injured or not during the two months prior to the race, and having the support or not of qualified staff for race preparation. Six hundred and twenty-four amateur level half-marathon athletes (515 men and 107 women; 41.5 ± 10.1 years) participated in the study. One week before competing in a half-marathon, participants answered the Hooper Index and the SF-36 questionnaire. Women stated higher stress before competing in the race (*p* < 0.01) compared to men and the group of runners of <40 years stated greater fatigue (*p* < 0.05) compared to the group of >40 years. Women showed a better quality of life in physical and emotional role dimensions (*p* < 0.05), and the group of >40 years showed a better quality of life in the emotional role dimension (*p* < 0.05). The group that had suffered an injury (InjuryYes) declared greater muscle soreness (MusclSore; *p* < 0.01), and the group that had qualified staff (QualifStaffYes) declared a higher level of stress (*p* < 0.05) and fatigue (*p* < 0.01). The Injury No (InjuryNo) group showed a better quality of life in the physical function dimension (*p* < 0.01). The group that did not have qualified staff (QualifStaffNo) showed a better quality of life in the dimensions of body pain, general health, vitality, social function (*p* < 0.05), and mental health (*p* < 0.01), while the QualifStaffYes group showed better results in the dimensions of physical function and emotional role (*p* < 0.05). Sex, age, being injured or not during the two months prior to the race, and having the support or not of qualified staff for the race preparation can influence the health and wellness status perception.

## 1. Introduction

Road running has evolved as an activity of increasing popularity [1], and almost every weekend races of this type take place worldwide. Specifically, the half-marathon is one of the most popular long-distance races as demonstrated by the 173 annual races homologated by the Royal Spanish Athletics Federation [2] and runners participating [3,4]. This type of races involves runners of different sex [5,6] and age [4] who are or are not injured [7], who train or do not train with qualified staff, who are highly trained and looking to improve their performance, or who are amateur runners (unprofessional) with a low level of training that simply aim to finish the race [4,8]. Despite the high participation of runners in this type of races [3] and that long-distance events are one of the most strenuous activities [9], the organizers of these events do not usually request any health check on runners as a requirement to participate [10]. It would be interesting to know, if the participants have adequate health perceptions to be able to make the efforts that are needed during the race, since if their perception is not good, runners, coaches, and event organizers could consider specific action strategies.

It may be difficult to screen the health of a large group of participants due to the costs, availability, accessibility, and time to objectively measure health, especially in the moments before the race. There are some validated self-administered questionnaires for this goal such as the SF-36 [11] or the Hooper Index [12]. The SF-36 measures the perception of the health of the participants, and the index proposed by Hooper et al. [12] measures the well-being ratings of athletes relative to stress, fatigue, muscle soreness, and sleep quality. These questionnaires have been used in other sport modalities, such as soccer [13,14,15], basketball [16], handball [17] or trail running [18], but we have not found any studies that analyze the health or the wellness status perception in amateur (non-professional) half-marathon runners before the competition. It would be interesting to know the health and wellness status perception of amateur half-marathon runners because, in many cases, they do not monitor and control their training and competition, and to make the relevant physical effort during this type of competition, they may need to have an adequate initial health status.

Therefore, in addition to ascertaining the perception of health and well-being in this group, it would be interesting to know if there are differences in the runners’ perception of health and wellness status according to their generic characteristics (sex, age (<40 and >40 years), whether they have been injured before or not, and whether they train with qualified coaches or not). This information could provide relevant data for coaches and runners about the perception of health and wellness status in half-marathon runners and the differences in these variables in the different population groups that take part in the races. Agrawal and D’Silva [19] obtained differences in the health and well-being perception according to sex; however, Batmyagmar et al. [20] did not find these differences based on sex in marathon runners. Valovich et al. [21] observed that the athletes who were injured declared lower values in the physical dimension compared to the non-injured athletes. Knowing whether athletes who have suffered an injury have a worse well-being and health perception or not could be interesting for studying the need to implement health-specific programs in injured athletes. Although there are some scientific studies in the literature, contradictory results have been found and, therefore, more studies may be necessary in this regard.

The aim of the present study was to analyze the wellness (i.e., stress, fatigue, muscle soreness, and sleep quality) and health status perception (i.e., physical function, physical role, body pain, general health, vitality, social function, emotional role, and mental health) in amateur half-marathon runners according to sex, age, having been injured or not during the previous two months, and having the support or not of qualified staff for race preparation prior to the competition. The hypothesis of the study was that the wellness and the health status perception before competing in a half-marathon could be different depending on sex, age, having been injured or not during the previous two months and having the support or not of qualified staff.

## 2. Materials and Methods

### 2.1. Sample and Participants

This study involved 624 amateur runners (41.5 ± 10.1 years) of whom 515 were men, 107 women, and two who were considered non-dichotomous and whose data were not considered for the analysis by sex. The runners were divided into two groups according to age: of all the runners participating in the study, 229 were under forty years old (<40 years; 30.7 ± 5.7 years) and 395 were over forty years old (>40 years; 47.7 ± 6.1 years). It was decided to take this cut-off age (<40 years old and >40 years old) into consideration according to the athletic racing regulations of the country where the study was conducted and the category classification used by the event organizers. One hundred and twelve participants had had an injury in the two months previous to participation in the race, and 512 had had no injury. One hundred and sixty-seven participants trained under the supervision of qualified staff and 457 did not. The inclusion criteria in the study were to have an official federative license, to be over 18 years old, and to have prepared to run in one of the following half-marathon races held in the Basque Country (Spain) in 2019: Behobia–San Sebastián, Zurich Marathon of San Sebastián (half-marathon modality), and/or Vitoria–Gasteiz Half-Marathon. Approval from the organizers of the indicated events was obtained before starting the study. Participant recruitment was carried out by the race organizers. Each organizer sent the questionnaire to all those who registered for the race and all the responses were collected from those runners who agreed to answer. There were no exclusion criteria; all of the responses to the questionnaire were accepted. All the participants were informed of the objectives of the study as well as the research procedure, and they voluntarily participated in it. The study followed the guidelines established in the Declaration of Helsinki (2013) and was approved by the Ethics Committee for Research with Human Beings (CEISH: M10_2019_243) of the University of the Basque Country (UPV/EHU).

### 2.2. Instruments and Procedures

The study was conducted from November 2019 to January 2020. The generic data from the runners were collected one week before participating in a half-marathon (Behobia–San Sebastián, Zurich Marathon in San Sebastián or Half-Marathon in Vitoria–Gasteiz). All study participants answered the parameters included in the Hooper et al. [12] study and the SF-36 questionnaire [11] on health-related quality of life anonymously. The participants provided their answers electronically.

### 2.3. Test

#### 2.3.1. Half-Marathon Runners Generic Data Collection

The generic data on all the runners were obtained with a questionnaire composed of 2 sections and 12 items: (1) 2 items (items 1–2) with short responses, referring to generic data, had to be completed by the runners (sex and age) and (2) 10 items (items 3–12) with a dichotomous response (yes or no) and multiple choice that referred to sport data (i.e., number of races contested during the year [N° races], having suffered an injury or not in the two months before the race that would have prevented the athletes from carrying out their usual or planned training for at least one week [InjuryYes and InjuryNo], preparation time for the race [RTPrep], average days/km per week of training during the previous six months [AverTrainDay and AverTrainKm], having qualified staff like a bachelor or graduate in Physical Activity and Sports Sciences, in Nutrition and Dietetics or any other type of qualified staff [QualifStaffYes] or not [QualifStaffNo] for the preparation of the race and type of qualification of the staff [QualifType]).

#### 2.3.2. Hooper Index

The Hooper Index, previously validated [12] and used with amateur athletes in the Spanish language [13,16,17], consisted of four items and was passed to all the participants with the purpose of assessing the perception of the level of stress (Stress), the state of fatigue (Fatigue), sleep quality (SleepQual), and muscle soreness (MusclSore) in the two weeks prior to participation in the race. The responses to the items were on a Likert-type scale (1 = very, very poor and 7 = very, very good) [21].

#### 2.3.3. SF-36 Health-Related Quality of Life Questionnaire

The SF-36 Health Questionnaire [11] is composed of 36 indicators that assess both positive and negative states of health. The 36 items in the instrument cover the following dimensions: physical function, physical role, body pain, general health, vitality, social function, emotional role and mental health. For the present investigation, a previously validated version was used in the two official languages of the autonomous community where the tests were held [22,23]. The responses to the items were on a Likert-type scale (1 to 5 with different responses depending on the question: 1 = excellent and 5 = poor, 1 = yes, it limits me a lot and 5 = no, it does not limit me at all, and 1 to 3 [items intense efforts [IntEff]—bathing or dressing by themself [BathDress]: 1 = Yes, it limits me very much and 3 = no, no limits for me). The responses given to each item were used as well as the sum of the items that make up each of the 8 dimensions analyzed in the questionnaire (physical function, physical role, body pain, general health, vitality, social function, emotional role and mental health). The dimensions were scored on a 0 (poor) to 100 (good health) [24].

### 2.4. Statistical Analysis

The results are presented as mean ± standard deviation (SD) or as frequencies or percentages of the total, for each of the answers provided by the participants in each item or question. Normal distribution and homogeneity of variances were tested using the Kolmogorov–Smirnov and Levene tests, and non-parametric statistical techniques were used. The internal consistency between the different items of the SF-36 questionnaire was evaluated using Cronbach’s alpha. The Mann–Whitney *U* Test was used to analyze the differences existing in the different dimensions of the Hooper Index and SF-36 questionnaire between the groups (men and women, runners <40 and >40 years, InjuryYes and InjuryNo, QualifSstaffYes, and QuialifStaffNo). In addition, the percentage of the mean difference (Dif.%) was calculated through the formula: Dif. (%) = [(Mean 2 − mean 1)/mean 1] × 100). The effect size (ES) was calculated according to the method proposed by Cohen [25]. Effect sizes less than 0.2, between 0.2 and 0.49, between 0.5 and 0.79, or greater than 0.8 were considered trivial, small, moderate, or large, respectively. Statistical analysis was performed with the statistical software IBM Statistical Package for Social Sciences Statistics, version 23.0 (IBM Inc, Armonk, NY, USA). The level of significance was set at *p* < 0.05.

## 3. Results

Table 1 shows the descriptive results of all the half-marathon amateur runners relative to the general data (generic data and sport data). The main profile of the amateur half-marathon runner was that of a man (82.5%), >40 years (63.3%), to have competed in 3.07 ± 0.92 races per year, to not have been injured during the two months prior to participation in the race (82.1%), and to not use qualified personnel to prepare for the event (83.2%). Participants in the study trained an average of approximately three days per week and had completed 36.17 ± 20.57 km of training per week for the six months prior to participating in the race.

The results for internal consistency showed adequate values for the set of items included in the SF-36 questionnaire (Cronbach’s Alpha = 0.71, *n* = 35 items), highlighting the total physical function (Cronbach’s Alpha = 0.91, *n* = 10 items), total physical role (Cronbach’s Alpha = 0.91, *n* = 4 items), and total emotional role (Cronbach’s Alpha = 0.94, *n* = 3 items) subscales. Table 2 shows the responses of the amateur half-marathon runners, as well as those obtained by the runners according to the sex and age of the participants (<40 and >40 years) on the Hooper Index and on the physical dimension of the SF-36 questionnaire. Regarding the results on the Hooper Index, women had higher stress than men before the race (Dif. = 12.5%, *p* < 0.01, small ES) and the group of runners of <40 years declared greater fatigue than the group of runners of >40 years (Dif. = −5.1%, *p* < 0.05, small ES). With respect to the variables on the physical dimension of the SF-36 questionnaire, women had lower scores in doing less than they would have liked because of physical health (PHDoLess) (Dif. = −3.9%, *p* < 0.05, small ES) compared to men. In addition, women showed a better quality of life in the dimensions of Physical role compared to men (Dif. = 7.1%, *p* < 0.05, small ES). According to the age of the runners, the group of <40 years declared better values in Health and health during the previous year (HealthPrevYear) (Dif. = 13.0% to 15.2%, *p* < 0.01, small ES), but showed worse results in reduced time spent at work or in daily activities because of physical health (PHRedTsDayAct), PHDoLess, difficulty doing a job or daily activities (PHDifficJob) (Dif. = −4.1% to 5.4%, *p* < 0.05 or *p* < 0.01, small ES) than the group of >40 years. The group of >40 years declared a greater limitation in performing intense efforts (IntEff) (Dif. = −1.8%, *p* < 0.05, trivial ES) compared to the group of <40 years. 

Table 3 shows the responses of the amateur half-marathon runners, as well as those obtained by the runners according to the sex and age of the participants (<40 and >40 years) on the Emotional dimension of the SF-36 questionnaire. Regarding the results on the variables of the Emotional dimension of the SF-36 questionnaire, women reduced time spent at work or in daily activities because of emotional problems (EPRedTsDayAct), doing less than they would have liked because of emotional problems (EPDoLess), doing things less carefully than they would have liked (EPLessCareful) (Dif. = −5.0%, *p* < 0.01, small ES), and they had lower scores in Nervous, LowMorale, discouraged and depressed (DiscourDepress), and Exhausted (Dif. = −3.7% to −6.3%, *p* < 0.05 or *p* < 0.01, small ES) compared to men. In addition, women showed a better quality of life in the dimensions of Emotional role compared to men (Dif. = 7.7%, *p* < 0.05, small ES). According to the age of the runners, the group of <40 years showed worse results in EPDoLess and, EPLessCareful, Nervous, LowMorale, Exhausted, Tired (Dif. = 3.3% to 9.7%, *p* < 0.05 or *p* < 0.01, small ES) than the group of >40 years. In addition, the group of >40 years showed a better quality of life in the Emotional role dimension compared to the group of <40 years (Dif. = 7.3%, *p* < 0.05, small ES).

Table 4 shows the results obtained by the amateur half-marathon runners depending on whether they had suffered an injury (InjuryYes) or not (InjuryNo) in the 2 months prior to participating in the race, as well as depending on whether they had qualified staff (QualifStaffYes) or not (QualifStaffNo) for the preparation of the race. Regarding the Hooper Index, the group of Injury runners declared greater MusclSore than those who had not suffered injury (Dif. = −20.7%, *p* < 0.01, moderate ES) and the group of runners QualifStaffYes declared a higher level of Stress and Fatigue compared to the group QualifStaffNo (Dif. = −7.5% to −7.8%, *p* < 0.05 or < 0.01, small ES). With respect to the Physical dimension variables of the SF-36 questionnaire, the group of runners InjuryYes showed worse results in Health, HealthPrevYear, IntEff, bending or kneeling (BendKneel), and PHRedTsDayAct (Dif. = −9.7% to 5.1%, *p* < 0.05 or *p* < 0.01, small ES) compared to runners InjuryNo. In addition, the Injury group did not show a better quality of life in the Physical Function dimension compared to the InjuryYes group (Dif. = 1.0%, *p* < 0.01, trivial ES).

The group of runners QualifStaffYes showed worse results in PHRedTsDayAct, PHDifficJob (Dif. = 2.8% to 3.2%, *p* < 0.05, small ES) compared to the QualifStaffNo group. By contrast, the QualifStaffNo group of runners declared greater difficulties for BendKneel because of physical health (PHBendKneel) (Dif. = −1.8%, *p* < 0.05, small ES) compared to the QualifStaffYes group. The QualifStaffNo group showed a better quality of life in the dimensions of Body Pain and General Health compared to the QualifStaffYes group (Dif. = 3.7% to 4.9%, *p* < 0.05, small ES), while the QualifStaffYes group did show better results in the Physical Function dimension (Dif. = −0.4%, *p* < 0.05, trivial ES).

Table 5 shows the results obtained by the amateur half-marathon runners depending on whether they had suffered an injury (InjuryYes) or not (InjuryNo) in the 2 months prior to participating in the race, as well as depending on whether they had qualified staff (QualifStaffYes) or not (QualifStaffNo) for the preparation of the race. Regarding the Emotional dimension variables of the SF-36 questionnaire, the group of runners QualifStaffYes showed worse results in EPRedTsDayAct, EPDoLess, EPLessCareful, Nervous, Exhausted and Tired (Dif. = 3.0% to 5.9%, *p* < 0.05 or *p* < 0.01, small ES) compared to the QualifStaffNo group. By contrast, the QualifStaffNo group of runners declared greater difficulties for reduced social activity because of physical health and/or emotional problems (PHEPActivSocial; Dif. = 3.0%, *p* < 0.05, small ES) compared to the QualifStaffYes group. The QualifStaffNo group showed a better quality of life in the dimensions of Vitality, Social Function, and Mental Health compared to the QualifStaffYes group (Dif. = 3.3% to 4.6%, *p* < 0.05 or *p* < 0.01, small ES), while the QualifStaffYes group did show better results in the Emotional Role dimension (Dif. = −8.5%, *p* < 0.05, small ES).

## 4. Discussion

The aim of the present study was to analyze the wellness (i.e., stress, fatigue, muscle soreness and sleep quality) and the health status perception (i.e., physical function, physical role, body pain, general health, vitality, social function, emotional role and mental health) in amateur half-marathon runners according to sex, age, being injured or not during the previous two months before the race, and having the support or not of qualified staff for the race preparation. The results obtained in the present study show that sex, age, having suffered an injury and the fact of training with qualified professionals can influence the health and wellness status perception in amateur runners at the moment before competing in a half-marathon. The novelty of this study was that this is the first investigation to analyze individual health perception before the race of amateur half-marathon runners. 

The Hooper Index (i.e., the amount of stress, fatigue, muscle soreness, and sleep quality) has been reported to be associated with athletes’ training loads [14,26,27]. In addition, the validity of the use of SF-36 health data has been demonstrated valid for measuring health indicators like physical function, physical role, body pain, general health, vitality, social function, emotional role, and mental health [11]. Mainly, the wellness index and the health measures have been focused on populations with disparate characteristics such as professional athletes, people with pathologies or youngsters [20,28,29,30]. However, scarce literature is available about amateur half-marathon runners. Considering the growth of the participation rates of different sex and age amateur runners in these races, and the scant control of health indicators at an amateur level in comparison with professionals, it would be interesting to know the health self-perception of the athletes not only as a general description but also distinguishing by sex and age [31]. Knowledge about the perception of the health of the amateur runners would allow us to propose specific actions, such as training with qualified staff as well as recommendations to avoid injuries, if necessary. In this respect, the main results of our study showed that women declared higher stress levels than men. Likewise, women perceived greater difficulties due to the fact of their physical state (i.e., PHDoLess, EPRedTsDayAct, EPDoLess, EPLessCareful; Dif. = −1.8% to 13%, *p* < 0.05 or *p* < 0.01, small ES) and greater difficulties based on emotional components (i.e., LowMorale, DiscourDepress, and Exhausted; Dif. = 4.4% to 9.7%, *p* < 0.05 or *p* < 0.01, small ES) the days before the competition in comparison to men. Nevertheless, women showed better values on the physical and emotional dimensions than men. Although Batmyagmar et al. [20] did not observe differences on the self-reported health measures depending on sex in elderly (i.e., over 60 years old) marathon runners, Agrawal and D’Silva [19] reported better values in Indian men versus women both on physical and mental dimensions. In this line of thought, other authors observed that women obtained worse health indicators than men [32]. The contradictory results found in the literature may be due to the differences in the participants’ ages and characteristics in the different studies (young versus older, athletes versus non-athletes), so more studies in this regard may be necessary. On the other hand, these results could be explained by social and biopsychosocial factors which could affect women’s self-esteem. As such, previous research has highlighted that the lower self-esteem in women could be possibly due to the work pathways across the life course as most women are responsible for caring for children, carrying out the home tasks, having difficulties to work outside the home with continuing employment being uneven and stalled [33,34] which can cause them to feel less valued and withdrawn from social life and lead to a worse perception of physical and mental health.

Regarding age, the half-marathon runners who were under 40 years old declared higher Fatigue than those athletes above 40 years old during the period prior to competition. In addition, athletes who were under 40 years old perceived greater difficulties due to the fact of their physical and emotional state (i.e., PHRedTsDayAct, PHDoLess, PhDifficJob, EPRedTsDayAct, EPDoLess and EPLessCareful) and showed higher levels in Nervous, LowMorale, Exhausted and Tired in comparison with athletes over 40 years old. Moreover, the athletes over 40 years old showed better quality of life values within the emotional dimension compared to athletes who were under 40 years old. Contrary to these findings, Agrawal and D’Silva [19] observed that people of the age group between 35 and 44 years old presented a better quality of life both in physical and mental dimensions, measured by the SF-36 health questionnaire, in comparison to older (over 45 years old) age groups. Surprisingly, our findings about the perception of Fatigue, as well as Physical and Emotional dimensions showed significantly lower values for half-marathon runners who were under 40 years old. These results could be explained by Waskiewicz et al. [4] as they reported associations between general motivation categories and the age of the athletes. As such, these authors observed that age was positively correlated with health orientation achievement, whereas personal goal achievement, competition, psychological coping, life meaning, and self-esteem were all negatively correlated with age [4]. Thus, it seems that while older marathon runners focus their objectives on health, the younger ones prioritize the objectives related to personal and competitive achievements and psychological aspects. This could cause higher pressure and, consequently, higher Fatigue perception and significantly lower self-reported health measures in the athletes who were under 40 years old. These results may highlight the need to analyze how runners of under 40 years prepare for competition and what factors influence having greater fatigue and a poor perception of health.

Acute and long-term sport injuries negatively affect athletes who suffer them, potentially lowering their quality of life and individual running performance due to the involvement of opposed emotions and inactive time periods [35]. In this respect, Valovich et al. [21] observed that those athletes who were injured at the time of completing the SF-36 declared lower values for physical functioning and limitations due to physical health problems, body pain, social function, and the physical dimension compared to the non-injured athletes. The results of our study showed that injured athletes presented higher pain levels and worse health self-perception values in comparison to non-injured ones. Likewise, injured athletes declared a feeling of worsening health compared to the last season, as well as limitations to perform IntEff and BendKneel. Additionally, injured athletes denoted a reduction on TsDayAct and lower values in the Physical function dimension compared to non-injured athletes. One of the possible causes may be that injuries normally cause tissues to tear, resulting in a less compliant area [36], and consequently increasing the pain sensation [21]. Furthermore, injuries can negatively affect race preparation, leading to psychological consequences (e.g., frustration, depression or tension) [37] which impact the athlete’s health [38]. Thus, training sessions should include strategies focused on reducing pain as well as implementing psychological interventions during rehabilitation programs [39], for example, self-regulation techniques (thought stopping, somatic relaxation, breathing) and stress management [40] or mindfulness [41]. These strategies have been shown to have positive effects in reducing the injury rate and also help to control psychosocial factors that increase the risk of injury [42]. The implementation of these strategies could be crucial to optimizing the return to the competition process, improving the athletes’ daily lives and reducing the re-injury risk, since to have suffered a previous injury is considered as the main risk factor for the occurrence of a new injury [43].

Although the quality of life based on health indicators has been analyzed for adolescent athletes and nonathletes [44], this is the first study differentiating the athletes depending on the qualified staff involved in the training process. Considering this, athletes who had the support of qualified staff (i.e., QualifStaffYes) during their preparation declared greater Stress and Fatigue levels compared to autonomous athletes (i.e., without support of qualified staff). Likewise, problems in Physical health and higher Nervous and Tired levels were reported by QualifStaffYes compared to QualifStaffNo. However, QualifStaffYes obtained better values in Physical function and Emotional role, and worse values in Body pain, General health, Vitality, Social function and Mental health dimension. On the other hand, QualifStaffNo presented greater limitations to BendKneel and in PHEPActivSocial compared to QualifStaffYes. Generally, athletes turn to qualified staff to optimize their preparation. However, this action seems to imply an increase in athletes’ stress levels [45], mainly due to the pressure that is felt by the athlete to comply with all scheduled workouts [46]. This stress can negatively impact on the athlete’s well-being due to the increased stress-related fatigue [47] as well as their general health, since psychological stress is closely related to a reduction of the immune function [48]. Additionally, the great amount of time required for high-intensity endurance training seems to negatively affect the time available for social contact [20], increasing athletes’ nervousness and fatigue. On the other hand, supervised training has been demonstrated to be safer and more effective than autonomous training in several populations [49,50]. This could explain why QualifStaffNo presented limitations such as BendKneel, since running implies a great demand on specific body structures (e.g., knee or Achilles tendon), which must be compensated for by specific training methods and recovery protocols. Those athletes QualifStaffNo would find it more difficult to apply the specific training methods and recovery protocols effectively. The results obtained in the present study suggest the importance of incorporating qualified staff such as bachelors or graduates in Physical Activity and Sports Sciences (PASS) or in Nutrition and dietetics during half-marathon preparation, mainly, to adjust the training periodization exercises and to reduce some negative emotional components [51].

This study is not without limitations. The first is that no information about the training strategies performed by the athletes (i.e., training contents, methodologies, and periodization) was reported, because it was considered that an excessively long questionnaire would have reduced the athletes’ response rate. Additionally, training load values (internal and/or external variables) were not collected. This information would make it possible to establish cause–effect relationships between the training process followed by amateur half-marathon athletes and their health-related quality of life. In this sense, future research lines should consider recording training contents, methodologies, and periodization. The second limitation is that no information was collected about the results obtained in the competition. Despite this, our study presents several strengths, highlighting the large sample (i.e., 624 amateur half-marathon participants in three different competitions) and the quality of life based on health indicators differentiating between sex, age, previously injured and non-injured athletes and the qualified staff involved in the training process.

## 5. Conclusions

The results obtained in the present study show that the health and wellness status perception is different depending on sex and age in amateur runners who compete in a half-marathon. In the same way, having suffered an injury and the fact of training with qualified professionals can influence the health and wellness status perception in amateur runners at the moment before competing in a half-marathon. Therefore, it would be interesting if both the organizers and the qualified personnel that collaborate in the preparation of amateur athletes took into account sex, age, and injury history in order to offer the best possible preparation to the athletes. 

The main findings of this study could be useful for the athletes, coaches and race organizers involved in the half-marathon context, to know how sex, age, having suffered an injury and training with qualified personnel affect the athletes’ well-being and health perception, in order to modify training strategies. For organizers of athletic competitions, these findings could provide them with the relevant data to know the athletes’ health self-perception and, consequently, to supply adequate recommendations based on health indicators before the race. It could be interesting for coaches to know the athlete’s well-being and health perception in order to be able to establish the objectives and the necessary training contents plan. This knowledge will allow trainers, on the one hand, to offer their professional services more appropriately, and on the other hand, to adjust the athletes’ training schedules and periodization. For runners, these data could be relevant to be aware of their individual initial state and health self-perception before the competition and, thereby, to request help, if necessary, to optimize their performance/health during races.

Thus, future studies should include training strategies performed by the athletes (i.e., training contents, methodologies and periodization) and the training load values (i.e., internal and/or external training load) in order to be able to analyze the influence of these variables on the health and well-being perception. Also, sport performance should be correlated with the perception of health, training hours and training load values. Likewise, it would be interesting to carry out similar studies with runners of other distances (i.e., marathon or ultra-marathon) as well as in other sport modalities (e.g., cyclists, mountain runners) in order to compare them with the results obtained in the present study.

## Figures and Tables

**Table 1:** Results of all the amateur half-marathon runners related to the generic and training data.

	All	Men	Women	Non-Dichotomous	<40 Years	>40 Years
***n* (%)**	624 (100%)	515 (82.5%)	107 (17.1%)	2 (0.3%)	229 (36.7%)	395 (63.3%)
**N° Races**	3.07 ± 0.92	3.10 ± 0.92	2.93 ± 0.95	2.50 ± 0.71	3.03 ± 0.98	3.09 ± 0.89
0–1 (%)	5.6%	5.4%	6.5%	0%	7.4%	4.6%
1–3 (%)	22.6%	21.2%	29%	50%	24%	21.8%
3–6 (%)	31.1%	31.5%	29%	50%	26.2%	33.9%
>6 (%)	40.7%	41.9%	35.5%	0%	42.4%	39.7%
**InjuryYes**	17.9%	17.9%	17.8%	50%	13.1%	20.8%
**PrePart**	69.1%	71.7%	56.1%	100%	52.9%	78.7%
**PreMark (min)**	97.58 ± 13.77	95.78 ± 13.35	108.84 ± 10.68	100 ± 14.14	95.66 ± 14.43	98.30 ± 13.46
**RTPrep (months)**	1.81 ± 1.19	1.80 ± 1.19	1.88 ± 1.20	1.50 ± 0.71	1.68 ± 1.10	1.88 ± 1.23
0–3 (%)	54.6%	55.5%	50.5%	50%	60.3%	51.4%
3–6 (%)	29%	30.8%	50%	50%	26.2%	30.6%
6–9 (%)	5.1%	7.5%	0%	0%	4.8%	5.3%
9–12 (%)	3.2%	2.8%	0%	0%	2.6%	3.5%
>12 (%)	8%	8.4%	0%	0%	6.1%	9.1%
**AverTrain** (Day)	3.57 ± 2.11	3.54 ± 2.25	3.74 ± 1.25	3.74 ± 1.25	3.66 ± 1.47	3.52 ± 2.41
**AverTrain** (Km)	36.17 ± 20.57	36.97 ± 21.17	32.54 ± 16.97	32.54 ± 16.97	34.95 ± 21.69	36.88 ± 19.88
**QualifStaff**	26.8%	26%	29%	100%	28.8%	25.6%
**QualifType**						
PASS (%)	69.6%	73.3%	54.8%	50%	69.7%	69.6%
NutritDiet (%)	6%	5.9%	3.2%	50%	4.5%	6.9%
Others (%)	24.4%	20.8%	42%	0%	25.8%	23.5%

<40 years = runners under 40 years old; >40 years = runners over 40 years old; N° Races = number of races contested per year; InjuryYes = having suffered an injury in the two months before the race; PrePart = previous participation in the race to be contested; PreMark = mark obtained in the previous edition; RTPrep = preparation time for the race; AverTrain = average days per week of training during the previous 6 months; QualifStaff = having qualified staff for the preparation of the race; QualifType = type of qualification of the staff for the preparation of the race; PASS = bachelor or graduate in physical activity and sports sciences; NutritDiet = bachelor or graduate in nutrition and dietetics; Others = other types of qualified staff.

**Table 2:** Results obtained for the Hooper Index and for the physical dimension of the SF-36 questionnaire obtained by the amateur half-marathon runners participating in the study and based on the sex and age of the participants (<40 and >40 years).

	All (*n* = 624)	Men (*n* = 515)	Women (*n* = 107)	Dif.% (ES)	<40 Years (*n* = 229)	>40 Years (*n* = 395)	Dif.% (ES)
**Hooper (0–7 scale)**							
Stress	3.42 ± 1.35	3.34 ± 1.36	3.76 ± 1.23	12.5 (0.3) **	3.38 ± 1.42	3.44 ± 1.32	1.7 (0.0)
Fatigue	3.55 ± 1.07	3.52 ± 1.06	3.67 ± 1.05	4.3 (0.1)	3.67 ± 1.14	3.48 ± 1.03	−5.1 (−0.2) *
SleepQual	3.32 ± 1.06	3.33 ± 1.04	3.21 ± 1.08	−3.6 (−0.1)	3.30 ± 1.16	3.33 ± 1.00	1.1 (0.0)
MusclSore	3.15 ± 1.13	3.13 ± 1.12	3.27 ± 1.18	4.6 (0.1)	3.17 ± 1.20	3.14 ± 1.09	−1.1 (0.0)
**SF-36 (1–5 scale)**							
HealthPrevYear	2.60 ± 0.77	2.60 ± 0.76	2.60 ± 0.76	−0.1 (0.0)	2.38 ± 0.86	2.74 ± 0.67	15.2 (0.5) **
IntEff (1–3 scale)	2.87 ± 0.36	2.87 ± 0.35	2.89 ± 0.35	0.7 (0.1)	2.90 ± 0.34	2.85 ± 0.37	−1.8 (−0.1) *
ModEff (1–3 scale)	2.96 ± 0.22	2.97 ± 0.20	2.96 ± 0.19	−0.1 (0.0)	2.95 ± 0.25	2.97 ± 0.19	0.5 (0.1)
ShopBag (1–3 scale)	2.97 ± 0.20	2.97 ± 0.19	2.95 ± 0.21	−0.7 (−0.1)	2.96 ± 0.23	2.97 ± 0.18	0.5 (0.1)
ClimbSevStair (1–3 scale)	2.95 ± 0.25	2.96 ± 0.23	2.93 ± 0.25	−0.7 (−0.1)	2.92 ± 0.31	2.96 ± 0.20	1.4 (0.2)
Climb1floor (1–3 scale)	2.97 ± 0.19	2.97 ± 0.19	2.98 ± 0.14	0.4 (0.1)	2.97 ± 0.21	2.97 ± 0.18	0.2 (0.0)
BendKneel (1–3 scale)	2.90 ± 0.31	2.90 ± 0.31	2.91 ± 0.29	0.1 (0.0)	2.92 ± 0.29	2.89 ± 0.32	−1.1 (−0.1)
WalkMore1km (1–3 scale)	2.96 ± 0.20	2.97 ± 0.20	2.97 ± 0.17	0.2 (0.0)	2.97 ± 0.17	2.96 ± 0.22	−0.2 (0.0)
WalkSevm (1–3 scale)	2.97 ± 0.20	2.97 ± 0.19	2.96 ± 0.19	−0.2 (0.0)	2.95 ± 0.23	2.97 ± 0.17	0.8 (0.1)
Walk100m (1–3 scale)	2.97 ± 0.19	2.97 ± 0.17	2.98 ± 0.14	0.3 (0.1)	2.96 ± 0.23	2.98 ± 0.17	0.7 (0.1)
BathDress(1–3 scale)	2.97 ± 0.21	2.97 ± 0.19	2.97 ± 0.17	0.0 (0.0)	2.96 ± 0.24	2.97 ± 0.18	0.5 (0.1)
Total Physical Function	97.4 ± 8.75	97.6 ± 8.6	97.6 ± 6.1	0.0 (0.0)	97.2 ± 7.9	97.5 ± 7.9	0.2 (0.0)
PHRedTsDayAct	4.69 ± 0.74	4.71 ± 0.70	4.62 ± 0.76	−2.1 (−0.1)	4.57 ± 0.82	4.76 ± 0.67	4.1 (0.3) **
PHDoLess	4.49 ± 0.85	4.53 ± 0.83	4.36 ± 0.87	−3.9 (−0.2) *	4.34 ± 0.95	4.58 ± 0.78	5.4 (0.3) **
PHDifficJob	4.73 ± 0.68	4.76 ± 0.63	4.65 ± 0.75	−2.2 (−0.2)	4.66 ± 0.75	4.77 ± 0.63	2.4 (0.2) *
PHDifficJobMoreCost	4.71 ± 0.68	4.73 ± 0.65	4.70 ± 0.69	−0.6 (0.0)	4.66 ± 0.72	4.74 ± 0.66	1.7 (0.1)
Total Physical Role	78.8 ± 33.5	77.7 ± 33.8	83.6 ± 31.9	7.1 (0.2) *	75.7 ± 35.8	80.6 ± 32.0	6.2 (0.2)
Pain	2.40 ± 1.15	2.37 ± 1.14	2.50 ± 1.17	5.2 (0.1)	2.41 ± 1.14	2.39 ± 1.16	−0.5 (0.0)
PainDifficJob	1.24 ± 0.54	1.22 ± 0.51	1.29 ± 0.55	5.9 (0.1)	1.27 ± 0.58	1.22 ± 0.51	−4.4 (−0.1)
Total Body Pain	78.2 ± 17.8	78.4 ± 18.2	77.7 ± 15.6	−0.9 (−0.0)	77.4 ± 20.7	78.7 ± 16.0	1.7 (0.1)
Health	2.26 ± 0.71	2.25 ± 0.71	2.29 ± 0.63	1.8 (0.1)	2.09 ± 0.71	2.36 ± 0.69	13.0 (0.4) **
Sickly	4.51 ± 0.83	4.52 ± 0.82	4.44 ± 0.87	−1.9 (−0.1)	4.43 ± 0.92	4.55 ± 0.77	2.8 (0.1)
Healthy	2.01 ± 1.10	2.00 ± 1.08	2.04 ± 1.14	2.0 (0.0)	2.08 ± 1.16	1.97 ± 1.06	−5.4 (−0.1)
HealthWorse	4.28 ± 0.99	4.27 ± 0.99	4.35 ± 0.95	1.7 (0.1)	4.29 ± 1.01	4.28 ± 0.98	−0.4 (0.0)
HealthExcell	1.88 ± 0.89	1.87 ± 0.87	1.96 ± 0.98	5.2 (0.1)	1.85 ± 0.92	1.90 ± 0.87	2.8 (0.1)
Total General Health	79.9 ± 14.1	80.1 ± 14.0	79.1 ± 14.8	−1.3 (−0.1)	79.0 ± 15.8	80.4 ± 13.1	1.7 (0.1)

Dif. (%) = average difference in percentage; ES = effect size; <40 years = runners under 40 years old; >40 years = runners over 40 years old; HealthPrevYear = health during the previous year; (difficulty for): IntEff = intense efforts; ModEff = moderate efforts; ShopBag = picking up or carrying a shopping bag; ClimbSevStair = climbing up several floors of stairs; Climb1floor = climbing up a single floor of stairs; BendKneel = bending or kneeling; WalkMore1km = walking a km or more; WalkSevm = walking several hundred meters; Walk100m = walking about 100 m; BathDress = bathing or dressing by themself; PH = physical health; RedTsDayAct = reduced time spent at work or in daily activities; DoLess = doing less than they would have liked; DifficJob = difficulty doing a job or daily activities; DifficJobMoreCost = difficulty doing a job or daily activities or costing more than normal; HealthWorse = health is going to get worse; HealthExcell = excellent health; Results are expressed as mean ± standard deviation. * *p* < 0.05 significant differences between means ** *p* < 0.01 significant differences between men and women or between <40 years and >40 years values.

**Table 3:** Results obtained for the Emotional dimension of the SF-36 questionnaire by the amateur half-marathon runners participating in the study and based on the sex and age of the participants (<40 and >40 years).

	All (*n* = 624)	Men (*n* = 515)	Women (*n* = 107)	Dif.% (ES)	<40 Years (*n* = 229)	>40 Years (*n* = 395)	Dif.% (ES)
**SF-36 (1–5 scale)**							
**Emotional Dimension**							
Vitality	2.26 ± 0.78	2.28 ± 0.78	2.16 ± 0.73	−5.3 (−0.2)	2.24 ± 0.85	2.27 ± 0.74	1.2 (0.0)
LotEnergy	2.32 ± 0.84	2.33 ± 0.85	2.25 ± 0.80	−3.4 (−0.1)	2.28 ± 0.90	2.34 ± 0.81	2.6 (0.1)
Exhausted	3.98 ± 0.94	4.03 ± 0.92	3.79 ± 0.97	−6.2 (−0.3) *	3.83 ± 0.88	4.08 ± 0.96	6.6 (0.3) **
Tired	3.65 ± 0.90	3.68 ± 0.91	3.56 ± 0.83	−3.2 (−0.1)	3.44 ± 0.90	3.77 ± 0.88	9.7 (0.4) **
Total Vitality	65.3 ± 12.4	65.1 ± 12.7	66.1 ± 11.4	1.5 (0.1)	65.1 ± 13.4	65.4 ± 11.9	0.4 (0.0)
PHEPActivSocial	1.36 ± 0.68	1.34 ± 0.65	1.47 ± 0.78	9.5 (0.2)	1.45 ± 0.77	1.31 ± 0.61	−9.5 (−0.2)
PHEPActivSocial	4.61 ± 0.71	4.63 ± 0.68	4.56 ± 0.79	−1.5 (−0.1)	4.56 ± 0.74	4.64 ± 0.70	1.7 (0.1)
Total Social Function	90.6 ± 15.5	90.7 ± 15.6	90.4 ± 14.8	−0.3 (−0.0)	89.0 ± 18.1	91.6 ± 13.6	2.8 (0.2)
EPRedTsDayAct	4.71 ± 0.69	4.75 ± 0.65	4.61 ± 0.70	−2.9 (−0.2) **	4.58 ± 0.88	4.79 ± 0.53	4.5 (0.3) *
EPDoLess	4.65 ± 0.73	4.70 ± 0.69	4.47 ± 0.76	−5.0 (−0.3) **	4.52 ± 0.88	4.72 ± 0.61	4.4 (0.3) **
EPLessCareful	4.66 ± 0.73	4.71 ± 0.70	4.48 ± 0.76	−4.9 (−0.3) **	4.53 ± 0.87	4.74 ± 0.61	4.6 (0.3) **
Total Emotional Role	78.5 ± 36.9	77.3 ± 37.2	83.8 ± 35.6	7.7 (0.2) *	74.8 ± 39.1	80.7 ± 35.5	7.3 (0.2) *
Nervous	3.91 ± 0.92	3.95 ± 0.91	3.70 ± 0.91	−6.3 (−0.3) **	3.70 ± 0.92	4.03 ± 0.90	8.7 (0.4) *
LowMorale	4.54 ± 0.80	4.57 ± 0.78	4.40 ± 0.85	−3.7 (−0.2) *	4.43 ± 0.86	4.60 ± 0.76	3.8 (0.2) **
CalmRelax	2.21 ± 0.89	2.20 ± 0.89	2.28 ± 0.89	3.5 (0.1)	2.20 ± 0.93	2.22 ± 0.86	1.0 (0.0)
DiscourDepress	4.34 ± 0.89	4.37 ± 0.88	4.20 ± 0.91	−4.1 (−0.2) *	4.25 ± 0.90	4.39 ± 0.88	3.3 (0.2) *
Happy	2.13 ± 0.87	2.12 ± 0.87	2.18 ± 0.83	2.6 (0.1)	2.09 ± 0.89	2.16 ± 0.87	3.6 (0.1)
Total Mental Health	69.7 ± 12.2	69.8 ± 12.3	69.8 ± 11.5	−0.0 (0.0)	69.4 ± 13.7	70.0 ± 11.3	0.8 (0.1)

Dif. (%) = average difference in percentage; ES = effect size; <40 years = runners under 40 years old; >40 years = runners over 40 years old; EP = emotional problems; LotEnergy = a lot of energy; PHEPActivSocial = reduced social activity because of physical health and/or emotional problems; RedTsDayAct = reduced time spent at work or in daily activities; DoLess = doing less than they would have liked; LessCareful = doing things less carefully than they would have liked; LowMorale = low morale; CalmRelax = calm and relaxed; DiscourDepress = discouraged and depressed. Results are expressed as mean ± standard deviation. * *p* < 0.05 significant differences between means ** *p* < 0.01 significant differences between men and women or between <40 years and >40 years values.

**Table 4:** Results obtained for the Hooper Index and for the Physical dimension of the SF-36 Questionnaire obtained by the amateur half-marathon runners participating in the study depending on whether they had suffered injury or not during the two months prior to participation in the race and depending on whether or not they had qualified staff for the preparation of the race.

	InjuryYes (*n* = 112)	InjuryNo (*n* = 512)	Dif.% (ES)	QualifStaffYes (*n* = 167)	QualifStaffNo (*n* = 457)	Dif.% (ES)
**Hooper (0–7 scale)**						
Stress	3.51 ± 1.46	3.40 ± 1.33	−3.2 (0.1)	3.62 ± 1.26	3.35 ± 1.38	−7.5 (−0.2) *
Fatigue	3.68 ± 1.10	3.53 ± 1.06	−4.2 (0.1)	3.77 ± 1.06	3.48 ± 1.07	−7.8 (−0.3) **
SleepQual	3.43 ± 1.10	3.29 ± 1.05	−3.9 (0.1)	3.35 ± 1.05	3.31 ± 1.06	−1.2 (0.0)
MusclSore	3.79 ± 1.17	3.01 ± 10.7	−20.7 (−0.7) **	3.29 ± 1.12	3.10 ± 1.14	−5.7 (−0.2)
**SF-36 (1–5 scale)**						
HealthPrevYear	2.78 ± 0.80	2.57 ± 0.75	−7.6 (0.3) *	2.59 ± 0.76	2.61 ± 0.77	0.7 (0.0)
**Physical dimension**						
EsfInt (1–3 scale)	2.77 ± 0.44	2.89 ± 0.33	4.4 (0.3) **	2.89 ± 0.35	2.86 ± 0.36	−1.1 (−0.1)
ModEff (1–3 scale)	2.95 ± 0.26	2.96 ± 0.20	0.6 (0.1)	2.96 ± 0.24	2.96 ± 0.21	−0.1 (0.0)
ShopBag (1–3 scale)	2.96 ± 0.21	2.97 ± 0.20	0.5 (0.1)	2.95 ± 0.24	2.97 ± 0.18	0.7 (0.1)
ClimbSevStair (1–3 scale)	2.94 ± 0.28	2.95 ± 0.25	0.4 (0.0)	2.95 ± 0.27	2.95 ± 0.25	−0.2 (0.0)
Climb1floor (1–3 scale)	2.97 ± 0.16	2.97 ± 0.20	−0.2 (0.0)	2.98 ± 0.19	2.97 ± 0.19	−0.3 (0.0)
BendKneel (1–3 scale)	2.84 ± 0.37	2.91 ± 0.29	2.6 (0.2) *	2.94 ± 0.26	2.89 ± 0.32	−1.8 (−0.2) *
WalkMore1km (1–3 scale)	2.96 ± 0.23	2.96 ± 0.19	0.0 (0.0)	2.97 ± 0.17	2.96 ± 0.21	−0.2 (0.0)
WalkSevm (1–3 scale)	2.98 ± 0.13	2.96 ± 0.21	−0.6 (0.1)	2.97 ± 0.20	2.97 ± 0.19	−0.1 (0.0)
Walk100m (1–3 scale)	2.97 ± 0.21	2.97 ± 0.19	−0.2 (0.0)	2.97 ± 0.20	2.97 ± 0.18	0.0 (0.0)
BathDress (1–3 scale)	2.97 ± 0.21	2.96 ± 0.20	−0.3 (0.0)	2.96 ± 0.26	2.97 ± 0.18	0.4 (0.1)
Total Physical Function	96.6 ± 8.2	97.6 ± 8.9	1.0 (0.1) **	97.7 ± 9.5	97.3 ± 8.5	−0.4 (0.0) *
PHRedTsDayAct	4.50 ± 0.94	4.73 ± 0.68	5.1 (0.3) **	4.59 ± 0.80	4.72 ± 0.71	2.8 (0.2) *
PHDoLess	4.34 ± 1.03	4.53 ± 0.81	4.3 (0.2)	4.41 ± 0.87	4.52 ± 0.85	2.4(0.1)
PHDifficJob	4.63 ± 0.82	4.75 ± 0.64	2.5 (0.2)	4.62 ± 0.82	4.77 ± 0.61	3.2 (0.2) *
PHDifficJobMoreCost	4.57 ± 0.90	4.75 ± 0.62	3.8 (0.2)	4.65 ± 0.76	4.74 ± 0.65	2.0 (0.1)
Total Physical Role	78.3 ± 33.3	78.9 ± 33.6	0.7 (0.0)	82.6 ± 30.8	77.4 ± 34.4	−6.7(−0.2)
Pain	3.30 ± 1.18	2.20 ± 1.05	−33.4 (1.0)	2.47 ± 1.16	2.37 ± 1.15	−3.9 (−0.1)
PainDifficJob	1.46 ± 0.71	1.19 ± 0.48	−18.4 (0.5)	1.29 ± 0.64	1.22 ± 0.49	−6.0 (−0.1)
Total Body Pain	79.7 ± 16.3	77.9 ± 18.1	−2.3 (−0.1)	75.3 ± 17.6	79.2 ± 17.8	4.9 (0.2) *
Health	2.46 ± 0.71	2.22 ± 0.70	−9.7 (0.3) **	2.23 ± 0.74	2.27 ± 0.70	2.1 (0.1)
Sickly	4.45 ± 0.94	4.52 ± 0.81	1.6 (0.1)	4.41 ± 0.95	4.54 ± 0.78	2.8 (0.1)
Healthy	2.08 ± 1.18	1.99 ±1.08	−4.2 (0.1)	1.97 ± 1.15	2.02 ± 1.08	2.7 (0.0)
HealthWorse	4.13 ± 1.11	4.32 ± 0.96	4.6 (0.2)	4.36 ± 0.97	4.25 ± 0.09	−2.4 (−0.1)
HealthExcell	2.03 ± 0.97	1.85 ± 0.87	−8.8 (0.2)	1.82 ± 0.93	1.90 ± 0.87	4.2 (0.1)
Total General Health	81.2 ± 12.5	79.6 ± 14.4	−1.9 (−0.1)	77.7 ± 14.7	80.7 ± 13.8	3.7 (0.2) *

Dif. (%) = average difference in percentage; ES = effect size; Injury(Yes/No) = having suffered an injury or not in the two months before the race that would have prevented the athlete from carrying out their usual or planned training for at least one week; QualifStaff(Yes/No) = having qualified staff or not for the preparation of the race HealthPrevYear = health during the previous year; IntEff = intense efforts; ModEff = moderate efforts; ShopBag = picking up or carrying a shopping bag; ClimbSevStair = climbing up several floors of stairs; Climb1floor = climbing up a single floor of stairs; BendKneel = bending or kneeling; WalkMore1km = walking a km or more; WalkSevm = walking several hundred meters; Walk100m = walking about 100 m; BathDress = bathing or dressing by themself; PH = physical health; RedTsDayAct = reduced time spent at work or in daily activities; DoLess = doing less than they would have liked; DifficJob = difficulty doing a job or daily activities; DifficJobMoreCost = difficulty doing a job or daily activities or costing more than normal; HealthWorse = health is going to get worse; HealthExcell = excellent health. Results are expressed as mean ± standard deviation. * *p* < 0.05 significant differences among means ** *p* < 0.01 significant differences between InjuryYes and InjuryNo or between QualifStaffYes and QualifStaffNo values.

**Table 5:** Results obtained for the Emotional dimension of the SF-36 Questionnaire obtained by the amateur half-marathon runners participating in the study depending on whether they had suffered injury or not during the two months prior to participation in the race and depending on whether or not they had qualified staff for the preparation of the race.

	InjuryYes (*n* = 112)	InjuryNo (*n* = 512)	Dif.% (ES)	QualifStaffYes (*n* = 167)	QualifStaffNo (*n* = 457)	Dif.% (ES)
**SF-36 (1–5 scale)**						
**Emotional Dimension**						
Vitality	2.35 ± 0.82	2.24 ± 0.77	−4.5 (0.1)	2.25 ± 0.82	2.27 ± 0.77	0.7 (0.0)
LotEnergy	2.39 ± 0.83	2.31 ± 0.85	−3.6 (0.1)	2.32 ± 0.91	2.32 ± 0.82	−0.1 (0.0)
Exhausted	3.89 ± 0.98	4.00 ± 0.93	2.9 (0.1)	3.87 ± 0.92	4.03 ± 0.94	4.1 (0.2) *
Tired	3.57 ± 0.91	3.67 ± 0.90	2.8 (0.1)	3.50 ± 0.90	3.71 ± 0.90	5.9 (0.2) **
Total Vitality	66.7 ± 11.1	64.9 ± 12.7	−2.8 (−0.1)	63.3 ± 12.4	66.0 ± 12.4	4.0 (0.2) *
PHEPActivSocial	1.45 ± 0.73	1.34 ± 0.66	−7.1 (0.1)	1.43 ± 0.71	1.34 ± 0.66	−6.1 (−0.1)
PHEPActivSocial	4.56 ± 0.86	4.62 ± 0.68	1.3 (0.1)	4.51 ± 0.81	4.65 ± 0.67	3.0 (0.2) *
Total Social Function	92.2 ± 13.0	90.3 ± 15.9	−2.1 (−0.1)	88.4 ± 16.9	91.4 ± 14.8	3.3 (0.2) *
EPRedTsDayAct	4.63 ± 0.82	4.73 ± 0.66	2.0 (0.1)	4.61 ± 0.79	4.75 ± 0.64	3.0 (0.2) *
EPDoLess	4.63 ± 0.83	4.66 ± 0.70	0.7 (0.0)	4.52 ± 0.88	4.70 ± 0.66	3.8 (0.2) *
EPLessCareful	4.60 ± 0.80	4.67 ± 0.71	1.6 (0.1)	4.56 ± 0.82	4.70 ± 0.69	3.0 (0.2) *
Total Emotional Role	78.9 ± 36.3	78.5 ± 37.1	−0.5 (0.0)	83.3 ± 33.5	76.8 ± 38.0	−8.5 (−0.2) *
Nervous	3.93 ± 0.93	3.90 ± 0.92	−0.7 (0.0)	3.76 ± 0.92	3.96 ± 0.92	5.2 (0.2) *
LowMorale	4.54 ± 0.76	4.54 ± 0.81	−0.2 (0.0)	4.49 ± 0.83	4.55 ± 0.79	1.3 (0.1)
CalmRelax	2.26 ± 0.91	2.21 ± 0.88	−2.4 (0.1)	2.24 ± 0.87	2.20 ± 0.89	−1.8 (0.0)
DiscourDepress	4.28 ± 0.90	4.35 ± 0.99	1.7 (0.1)	4.29 ± 0.89	4.35 ± 0.89	1.4 (0.1)
Happy	2.32 ± 1.01	2.09 ± 0.84	−9.8 (0.2)	2.06 ± 0.88	2.16 ± 0.87	4.8 (0.1)
Total Mental Health	70.1 ± 11.2	69.7 ± 12.4	−0.6 (0.0)	67.3 ± 12.7	70.6 ± 12.0	4.6 (0.3) **

Dif. (%) = average difference in percentage; ES = effect size; QualifStaff = having qualified staff for the preparation of the race; EP = emotional problems; LotEnergy = a lot of energy; PHEPActivSocial = reduced social activity because of physical health and/or emotional problems; RedTsDayAct = reduced time spent at work or in daily activities; DoLess = doing less than they would have liked; LessCareful = doing things less carefully than they would have liked; LowMorale = low morale; CalmRelax = calm and relaxed; DiscourDepress = discouraged and depressed. Results are expressed as mean ± standard deviation. * *p* < 0.05 significant differences among means ** *p* < 0.01 significant differences between InjuryYes and InjuryNo or between QualifStaffYes and QualifStaffNo values.
